# Case Report: Perioperative management of a pregnant poly trauma patient for spine fixation surgery

**DOI:** 10.12688/f1000research.6659.2

**Published:** 2015-07-24

**Authors:** Rashmi Vandse, Meghan Cook, Sergio Bergese

**Affiliations:** 1 Department of Anesthesiology, Wexner Medical Center, Ohio State University, Columbus, Ohio, 43210, USA

**Keywords:** Spine surgery, pneumothorax, neuro anesthesia, pregnant, poly trauma

## Abstract

Trauma is estimated to complicate approximately one in twelve pregnancies, and is currently a leading non-obstetric cause of maternal death. Pregnant trauma patients requiring non-obstetric surgery pose a number of challenges for anesthesiologists. Here we present the successful perioperative management of a pregnant trauma patient with multiple injuries including occult pneumothorax who underwent T9 to L1 fusion in prone position, and address the pertinent perioperative anesthetic considerations and management.

## Introduction

Perioperative management of a pregnant patients requiring non-obstetric surgery is always challenging for an anesthesiologist. The literature documenting anesthetic, surgical and obstetric management of pregnant poly trauma victims undergoing spine surgery in prone positioning is limited. We present a case of a pregnant polytrauma victim with multiple injuries who subsequently underwent spine fixation surgery in prone position and discuss pertinent anesthetic issues and management.

## Case presentation

**Figure 1.**
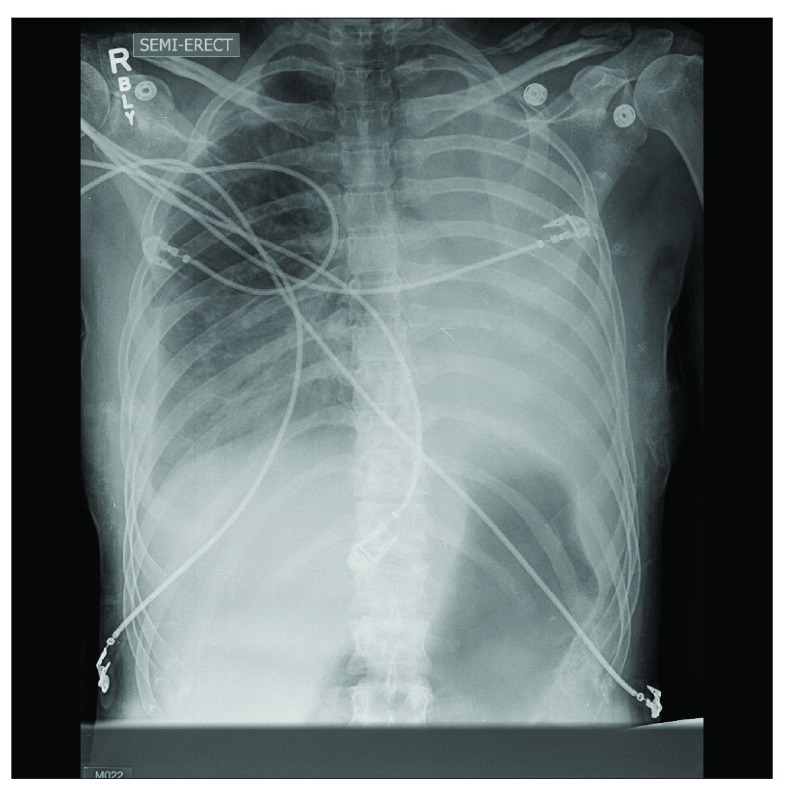
A preoperative chest X-ray showing complete collapse of the left lung.

This is a case of a previously healthy 32 year old female, who presented while 17 weeks pregnant as a level 2 trauma following a motor vehicle collision. She had sustained multiple injuries including Grade II liver laceration, pelvic fracture, bilateral clavicle fractures, C1 transverse process fracture, T11 vertebral body burst fracture, R rib 1–10 fractures, L 1st and 2nd rib fractures, bilateral small pneumothoraces and right pulmonary contusion. She was moderately built and nourished, was 66 inches tall and weighed 136 pounds. Her vital signs on admission showed: heart rate of 96 beats/minute, respiratory rate of 14–18 breaths/minute, blood pressure of 108/56 mmHg, and O2 saturation of 98% on 2–3 liters of oxygen through nasal cannula. She remained hemodynamically stable throughout and did not show any signs of respiratory distress, although she did have some trouble with coughing and clearing respiratory secretions. A preoperative chest X-ray demonstrated complete collapse of the left lung ( Figure 1). The small pneumothorax that was discovered in a computed tomography (CT) of the chest, however, was not apparent in the chest X-ray. After a multidisciplinary discussion, because of the unstable spine fracture, it was decided to perform a posterior T9-L1 fusion under general anesthesia. Her lab values were otherwise normal except for hemoglobin of 9.5 and hematocrit of 27.4.

General anesthesia was induced with propofol, lidocaine, fentanyl and succinylcholine. Following intubation, bronchoscopy was performed and the airway was suctioned given her preoperative chest X-ray. The radial artery was cannulated for hemodynamic monitoring. She was then carefully positioned prone on an open frame Jackson table. Special care was taken to avoid any pressure on the abdomen and all the other pressure points were checked and padded. Anesthesia was maintained with propofol (50 mcg/kg/min) and remifentanil (0.05–0.12 mcg/kg/min) infusions along with 1.0% sevoflurane in 50% oxygen. Phenylephrine was used to support her blood pressure as needed. She remained hemodynamically stable throughout the procedure. She was ventilated with a small tidal volume (300–350 ml) and her peak pressure was closely monitored, which stayed less than 20 cm of H20 throughout. CT-based image guidance was mostly used by the surgeons to limit the intraoperative fluoroscopy. She received 1300 ml of crystalloids and 500 ml of albumin. She produced 400 ml of urine and lost approximately 200 ml of blood. Total duration of anesthesia was approximately 4 hours. She was successfully extubated at the end of the procedure. She remained stable post operatively. However, she did require 22 days of inpatient care due to multiple injuries sustained during the trauma. She was successively discharged home. She later came back at term and delivered a healthy baby by elective Caesarean section under general anesthesia.

## Discussion

Trauma is estimated to complicate approximately one in twelve pregnancies, and is currently a leading non-obstetric cause of maternal death; moreover, maternal death remains the most common cause of fetal demise ^1– 4^. Extensive multidisciplinary planning between the surgeons, intensivists, anesthesiologists and obstetricians is essential to ensure fetal and maternal well-being throughout the perioperative period. The anesthetic considerations of this case were many. We had a pregnant patient requiring spine fixation surgery in prone position. Her management was further convoluted by the associated injuries, most importantly b/l rib fractures and small pneumothoraces.

## Obstetric concerns

Optimum management requires a thorough understanding of normal maternal-fetal physiology, maternal physiologic adaptation to pregnancy and altered drug pharmacodynamics and pharmacokinetics. The increased oxygen requirements, decreased functional residual lung capacity and increased risk for aspiration associated with pregnancy complicates perioperative management by decreasing the time available and the margin of safety. These changes are extensively reviewed in many textbooks and review articles ^5– 7^. The gestational age and maturity of the fetus as well as the acute maternal injuries were taken into account when formulating the operative plan. As the patient was in her second trimester, delivery of the fetus was not a feasible option. The fetal heart tones (FHT) were monitored pre and post operatively, which remained stable.

The deleterious effects of anesthesia on the human fetus have been considered for many years. As such, any drug has the potential to negatively affect the developing human fetus depending on the dose and the time of exposure and there is no “ideal anesthetic agent” ^5– 8^.

It is therefore most prudent to postpone elective surgical procedures until after pregnancy or, if possible, to avoid during the first trimester ^5, 6^. There is no convincing evidence that any particular anesthetic drug at clinically used doses is clearly dangerous to the human fetus ^5, 6^. Anesthetic goals are to prevent fetal asphyxia by maintaining maternal oxygenation, ventilation and hemodynamic stability and avoid factors that might cause reduction in the uteroplacental perfusion or compromise fetal gas exchange ^5– 7^. Large survey studies on women who underwent surgery during pregnancy suggest no increase in congenital anomalies among their offspring but rather an increase in the risk for abortions, growth restriction for reasons mostly attributed to the requirement for surgery but not anesthetic administration ^5, 6^. In our patient, we used a combination of intravenous (IV) anesthetics (propofol and remifentanil) with 1% sevoflurane in order to permit Somatosensory Evoked Potential (SSEP) and electromyogram (EMG) monitoring and allowed for rapid awakening at the end of the procedure. In clinical practice both propofol and remifentanil have been used safely in pregnant patients ^10– 13^. Caution must be exercised while using propofol infusion for long procedures (>10 hours). Two cases of prolonged IV anesthesia with propofol during pregnancy (14–18 h) resulted in mild metabolic acidosis ^14^. Maintenance of normal maternal blood pressure is imperative because of the relative passive dependence of the uteroplacental circulation and also to avoid spinal cord ischemia. As such a reduction in maternal arterial pressure causes reduced uteroplacental blood flow and fetal ischaemia. We used phenylephrine infusion to maintain MAP above 70 mmHg based on the earlier studies supporting better maternal cardiovascular stability and improved neonatal acid–base status when phenylephrine was used to treat maternal hypotension ^5, 6^.

## Surgical positioning

Some of the case reports and small case series have described good fetal outcome among gestational women who had spinal surgery during their pregnancy ^15– 19^. Caution must be exercised while positioning the patient to avoid any compression on the gravid uterus. Aortocaval compression must be avoided as this can lead to significant reductions in maternal cardiac output, systemic blood pressure, and uterine blood flow. This can also cause epidural venous engorgement and increased surgical bleeding. The study by Nakai *et al.* ^20^ showed that when pregnant patients were positioned prone by letting the abdomen hang free, there was actually better relief of compression on the large maternal vessels by the gravid uterus when compared to sitting or lateral positions. We used a Jackson frame, which helped to avoid any direct compression of the fetus and the great vessels. One of the drawbacks of prone positioning is inability to easily monitor fetal status or perform emergent cesarean section for fetal distress in a viable fetus. Spinal surgeries have been performed successfully under epidural anesthesia and lateral positioning has also been utilized safely during the late second and third trimester of pregnancy ^15^. These alternative surgical approaches must be discussed with the surgeon whenever feasible in patients with a viable fetus. 

## Rib fractures and pneumothorax

The presence of multiple b/l rib fractures and b/l occult (small) pneumothorax impacted our decision making because of the increased risk for expanding the pneumothorax with positive pressure ventilation. The management of an occult or clinically insignificant pneumothorax in acute trauma patients is controversial. The development of tension pneumothorax intraoperatively requiring emergency chest tube insertion has been reported ^21^. In a prospective randomized study by Enderson *et al.*, 8 out of 21 patients in the observation group demonstrated progression of the occult pneumothorax and 3 of them developed a tension pneumothorax ^22^. They suggested that mechanically ventilated patients with an occult pneumothorax should be managed with a thoracostomy tube. On the contrary, there appears to be a growing recognition that vast majority of cases with an occult pneumothorax can be safely treated without placing a thoracostomy tube in non-ventilated or even mechanically ventilated patients ^23– 26^. Hence in the absence of clear-cut evidence one must consider the risk versus benefit while making the clinical decision. Thoracostomy is also associated with major complications and has been reported to increase the overall mortality rate ^25, 26^. As such, in this case, special attention was paid to the peak airway pressures and plethysmography. Additionally, the general surgery team was made aware of the patient, and a chest tube kit was kept in the room, although it was not needed during this case. Our patient also had decreased aeration on the L side of her lung in the preoperative chest X-ray which was thought to be due to an inability to clear the secretions as a result of splinting. Flexible bronchoscopy and aspiration of secretion was performed after intubation. Postoperatively, meticulous attention was paid to adequate pain control and incentive spirometry, which allowed further improvement in the lung aeration. She was also placed on intermitted BiPAP as needed. Thus, we were able to avoid prolonged intubation as well as chest tube insertion.

## Radiation exposure

Additional consideration was also given to radiation exposure. Radiographic studies have shown that radiation exposure poses the greatest teratogenic risk in early pregnancy when organogenesis occurs (2–7 weeks) ^5, 9^. Exposure after organogenesis may cause growth restriction, microcephaly, and childhood cancer ^5, 9, 27^. Fetal risk of malformations is considered to be low with total radiation exposures of less than 50 to 100 mGy (5 to 10 rads) ^27^. In contrasts to the negligible risk of teratogenicity, observational studies suggest that there is a slightly higher risk of childhood cancer at radiation doses greater than or equal to 10 mGy ^28^. Therefore, exposure to radiation should be minimized whenever possible. Computed tomography produces higher levels of radiation exposure than plain radiographs, but even abdominal and pelvic CT scanning usually produces estimated fetal exposures below those typically associated with adverse fetal/neonatal outcomes ^5, 28^. In our case, CT-base image guidance was mostly used by the surgeons to limit the intraoperative fluoroscopy.

## Conclusion

Successful surgical intervention was accomplished without any major morbidity or mortality due to thorough systematic assessment of individual issues and stratification of management priorities. The ultimate objective is to provide safe anesthesia to the mother while concurrently minimizing the risk of preterm labor or fetal demise. In our case, the patient was successively discharged home and delivered a healthy baby at term without any complications.

## Consent

Written informed consent for publication of their clinical details and/or clinical images was obtained from the patient.
