# Supplementary material for: Case Report: Perioperative management of a pregnant poly trauma patient for spine fixation surgery
Source: F1000Res. 2015 Jul 24;4:171. Originally published 2015 Jun 29. [Version 2] doi: 10.12688/f1000research.6659.2 (PMC4536612; doi:10.12688/f1000research.6659.2)
Supplement: Peer review history

# Peer review: Referee response for version 2

**DOI:** 10.5256/f1000research.7368.r9715

**Published:** 2015-07-30

**Authors:** Michael Paech

**Affiliations:** 1 School of Medicine and Pharmacology, University of Western Australia, Perth, WA, Australia

I am happy to approve the revision of the case report without further comment.

I have read this submission. I believe that I have an appropriate level of expertise to confirm that it is of an acceptable scientific standard.

# Peer review: Referee response for version 2

**DOI:** 10.5256/f1000research.7368.r9647

**Published:** 2015-07-28

**Authors:** Bernard Wittels

**Affiliations:** 1 Continental Anesthesia LLC, Saint Francis Hospital, Evanston, IL, USA

The authors have revised their manuscript in a manner that addresses the concerns of this reviewer, including the mention of alternative surgical approaches and the discussion of the relative benefits of their choice of anesthetic care.  Further work in this area of neurosurgery during pregnancy may lead to more rigorous analysis and safer practices.

I have read this submission. I believe that I have an appropriate level of expertise to confirm that it is of an acceptable scientific standard.

# Peer review: Referee response for version 1

**DOI:** 10.5256/f1000research.7153.r9227

**Published:** 2015-07-14

**Authors:** Bernard Wittels

**Affiliations:** 1 Continental Anesthesia LLC, Saint Francis Hospital, Evanston, IL, USA

This is a report of an exceptionally unusual presentation of a parturient with multiple trauma needing urgent neurosurgery. The authors do a thorough review of important aspects of parturient physiology, as well as the medical and surgical considerations involved. Still, the authors seem to have followed the typical care of any neurosurgical patient for this procedure and were just serendipitous in having a Jackson-type operating table with an open suspended sling that prevented any untoward pressure on the fetus or fetal circulation while in the prone position. If a more unique approach to neurosurgery or neuroanesthesia in the parturient was introduced and proven successful, this report would have much greater value to its readers.

The main scientific point is that the positioning and monitoring of a non-pregnant patient for neurosurgery serves well for the polytrauma pregnant patient for many reasons as well described, though not directly.  Also missing is the inability to easily monitor the fetal status intraoperatively, and the inability to perform emergent cesarean delivery in this position if fetal distress occurs in a viable fetus.  Are there modifications in the anesthetic or surgical approach that could accommodate these factors?

I have read this submission. I believe that I have an appropriate level of expertise to confirm that it is of an acceptable scientific standard, however I have significant reservations, as outlined above.

# Response

**Published:** 2015-07-18

**Authors:** Rashmi Vandse

**Affiliations:** Wexner medical center at ohio state university, USA

We thank our reviewer for their input. We have modified the case report as suggested.

As the patient was in her early pregnancy, we did not have significant challenge in positioning her prone. Also since the fetus was non viable we didn’t encounter any need to modify her positioning.  As suggested in the literature, surgery in prone position has been carried out safely during the first and early second trimester.  As pointed out by the reviewer , one of the drawbacks of prone positioning is inability to monitor fetal status or perform emergent cesarean section for fetal distress in a viable fetus. Spinal surgeries have been performed successfully under epidural anesthesia and lateral positioning has also been utilized safely during the late second and third trimester of pregnancy ^15^.  These alternative surgical approaches must be discussed with the surgeon whenever feasible.

# Peer review: Referee response for version 1

**DOI:** 10.5256/f1000research.7153.r9420

**Published:** 2015-07-10

**Authors:** Michael Paech

**Affiliations:** 1 School of Medicine and Pharmacology, University of Western Australia, Perth, WA, Australia

This is a case report with an extended discussion of perioperative management in this setting. Although the patient was pregnant, she was in the early second trimester, when the relevance of some physiological changes of pregnancy and the absence of a distended abdomen or a viable fetus mean that the management is mainly specific to trauma issues - pregnancy issues are not well illustrated.  Consequently the educational value of this report is diminished.

My main suggestions are:

1. Delete the sentence in the case presentation “On examination…..”
2. Add a sentence as to whether an attempt made to confirm fetal viability before and after surgery, and by what method.
3. The Discussion section on “obstetric concerns” is too long, a regurgitation of standard teaching and is not specific to this patient. This should be shortened. Likewise much of the first half on “surgical positioning” is not relevant to this patient.
4. Please check for quality of language. Examples of poor use include “of a pregnant patients” ; “the obstetric team was consulted who…” ; “multiple b/l rib……”; “3 of which…”

I have read this submission. I believe that I have an appropriate level of expertise to confirm that it is of an acceptable scientific standard, however I have significant reservations, as outlined above.

# Response

**Published:** 2015-07-18

**Authors:** Rashmi Vandse

**Affiliations:** Wexner medical center at ohio state university, USA

We would like to thank our reviewer for reviewing our case report.  We have modified it as per the suggestion. As pointed out by the reviewer, our patient was in her early 2 nd trimester hence lacked the viable fetus or the distended abdomen. Physiologic changes of pregnancy are less pronounced. However as suggested in the literature, surgery during the early pregnancy carry increased risk for miscarriage and it is important to take all precautions to maintain adequate maternal oxygenation and hemodynamic stability which was complicated in our patient due to her polytrauma status.

 

1. *Delete the sentence in the case presentation “On examination…..”-   modified*
2. *Add a sentence as to whether an attempt made to confirm fetal viability before and after surgery, and by what method.- FHT were monitored both pre and post operatively which was reassuring.*
3. *The Discussion section on “obstetric concerns” is too long, a regurgitation of standard teaching and is not specific to this patient. This should be shortened. Likewise much of the first half on “surgical positioning” is not relevant to this patient.- Has been shortened.  However some of the important obstetric issues have been retained for educational purposes. Surgical positioning – Has been shortened. *
4. *Quality of language has been checked and corrections have been made. *
